# Systematic identification of cell cycle regulated transcription factors from microarray time series data

**DOI:** 10.1186/1471-2164-9-116

**Published:** 2008-03-03

**Authors:** Chao Cheng, Lei M Li

**Affiliations:** 1 Molecular and Computational biology program, Department of Biological Sciences, University of Southern California, Los Angeles, CA 90089-2910, USA; 2 Department of Mathematics, University of Southern California, Los Angeles, CA 90089, USA

## Abstract

**Background:**

The cell cycle has long been an important model to study the genome-wide transcriptional regulation. Although several methods have been introduced to identify cell cycle regulated genes from microarray data, they can not be directly used to investigate cell cycle regulated transcription factors (CCRTFs), because for many transcription factors (TFs) it is their activities instead of expressions that are periodically regulated across the cell cycle. To overcome this problem, it is useful to infer TF activities across the cell cycle by integrating microarray expression data with ChIP-chip data, and then examine the periodicity of the inferred activities. For most species, however, large-scale ChIP-chip data are still not available.

**Results:**

We propose a two-step method to identify the CCRTFs by integrating microarray cell cycle data with ChIP-chip data or motif discovery data. In *S. cerevisiae*, we identify 42 CCRTFs, among which 23 have been verified experimentally. The cell cycle related behaviors (e.g. at which cell cycle phase a TF achieves the highest activity) predicted by our method are consistent with the well established knowledge about them. We also find that the periodical activity fluctuation of some TFs can be perturbed by the cell synchronization treatment. Moreover, by integrating expression data with in-silico motif discovery data, we identify 8 cell cycle associated regulatory motifs, among which 7 are binding sites for well-known cell cycle related TFs.

**Conclusion:**

Our method is effective to identify CCRTFs by integrating microarray cell cycle data with TF-gene binding information. In *S. cerevisiae*, the TF-gene binding information is provided by the systematic ChIP-chip experiments. In other species where systematic ChIP-chip data is not available, in-silico motif discovery and analysis provide us with an alternative method. Therefore, our method is ready to be implemented to the microarray cell cycle data sets from different species. The C++ program for AC score calculation is available for download from URL .

## Background

Eukaryotic cell cycle is precisely controlled and regulated at the transcriptional, post-transcriptional, and post-translational level. To identify cell cycle regulated genes, several genome-wide analysis have been performed using microarray technologies [1-5]. In these studies, expression levels across the cell cycle were measured simultaneously for thousands of mRNA transcripts. In order to identify the subset of periodically expressed genes in the resulting microarray gene expression time series data, a number of computational approaches have been proposed, including Fourier analysis [2,6], partial least squares regression [7], Fisher's G-test [8], model-based method [9], and methods using some threshold criteria [10]. These approaches provided useful tools for periodicity analysis in microarray time series data and have led to the identification of hundreds of cell cycle regulated genes. For example, Spellman *et al*. found that about 800 genes are periodically expressed across the cell cycle in *S. cerevisiae*.

Transcription factors (TFs) play critical roles in gene expression regulation. To understand how the cell cycle is regulated and how cell cycle regulates other biological processes, such as DNA replication and amino acids biosynthesis, it is useful to identify the cell cycle regulated transcription factors (CCRTFs). We note that in this paper we use the term "cell cycle regulated" rather than "cell cycle regulator" as used in previous studies, because it is often difficult to infer the direction of regulation only from the microarray cell cycle data. The transcription factors whose regulatory activities fluctuate periodically across the cell cycle could be either cell cycle regulator or effector of the cell cycle regulation. Moreover, the expression levels of TFs in microarray data may not accurately reflect their activities in transcription regulation. First, TFs are often subject to various post-transcriptional and post-translational modifications, which abolish the significant correlations between their activities and expression levels. Second, TFs are usually expressed in relatively low levels [11,12] and therefore expression changes measured by microarray hybridization may not be accurate. Therefore, identification of CCRTFs should be based on the activities of TFs rather than their expression levels. Considering this issue and by integrating the microarray cell cycle data with TF-gene connectivity data from ChIP-chip experiment, several methods have been suggested to identify yeast cell cycle transcription factors [13-15], to infer cooperativity among the transcription factors controlling the cell cycle in yeast [16], to model the network of yeast cell cycle transcription factors [17], or to reconstruct the transcriptional regulatory modules of the yeast cell cycle [18].

In this paper, we suggest a two-step method to identify the CCRTFs in yeast. First, for each TF we infer its activity in each time point of the microarray data, resulting in an activity profile for it. This is achieved by integrating microarray expression data with systematic ChIP-chip data [19] or motif discovery data [20] using the BASE method [21]. In the second step we use the Fisher's G-test to examine the periodicity of these TF activity profiles to identify the CCRTFs. Based on the combination of microarray data and ChIP-chip data, we identify 42 CCRTFs at 4% false discovery rate (FDR), including 23 experimentally validated cell cycle TFs. More importantly, by combining microarray expression data with in-silico motif discovery data, we identify 8 motifs with known associated TFs at 3.5% FDR, among which 7 are associated with established cell cycle TFs. Thus, our method is able to identify CCRTFs in species other than S. cerevisiae, in which microarray cell cycle data have been generated whereas large-scale ChIP-chip data are still not available.

## Results

### Periodic activity fluctuation of TFs across the cell cycle

We integrate the microarray cell cycle gene expression data with the ChIP-chip data to infer TF activities at 18 time points of the microarray data, resulting in 203 activity change score (AC score) profiles each corresponding to a TF. These AC score profiles measure the activity fluctuation of TFs at each of the 18 time points in the cell cycle. The AC score for a TF reflects the relative activity of this TF in the synchronized sample with respect to the non-synchronized control at a time point. For a transcription activator, a positive AC score indicates its activity enhancement and a negative AC score indicates its activity reduction. For a transcription repressor, the inverse conclusion should be made. See section "Methods" for detail definition and calculation of AC scores. Then average periodogram is applied to these AC score profiles and the original gene expression data (only the expressions for TF coding genes are selected from the microarray data) to investigate the existences of TFs that fluctuate periodically in the activity and in the expression level, respectively.

**Figure 1:**
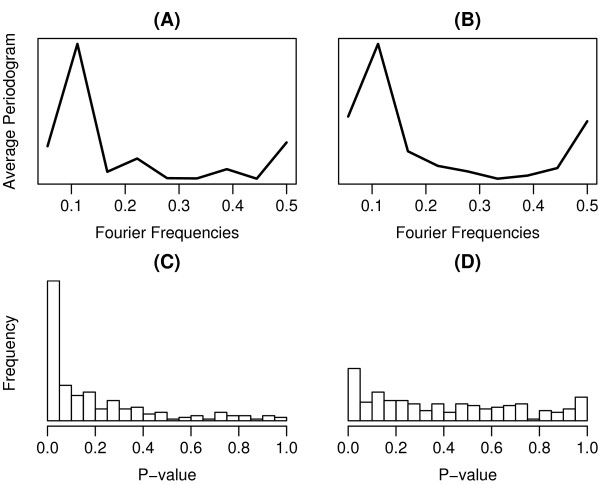
**Periodicity of TF activities and expression levels across the cell cycle**. The average periodograms for inferred TF AC score data and TF expression data are shown in (A) and (B), respectively. The Fisher's G-tests are applied to each of the 203 TF AC score profiles and expression profiles. Distributions of the resulting p-values are shown in (C) and (D), respectively.

As shown in Figure 1, the average periodogram exhibits a dominate peak at the Fourier frequency of 0.11 in both the inferred AC score data (Figure 1A) and the gene expression data (Figure 1B), suggesting the existences of periodical components. The Fourier frequency 0.11 corresponds to the genuine frequency of the cell cycle, since the microarray data covers exactly two cell cycles by 18 time points. These results indicate that the existences of the cell cycle regulated TFs can be detected at both the activity level and the expression level. Nevertheles, when we test the significance of periodicity for each of the TFs using the Fisher's G-test, we find that much more TFs show periodic fluctuation in the activity level (Figure 1C) than in the gene expression level (Figure 1D). At the 0.01 significance level, we identify 42 CCRTFs based on the inferred TF activity profiles, whereas only 4 TFs are found to be cell cycle regulated based on the original expression data. These results confirm our hypothesis that cell cycle regulation of TFs takes place mainly at the activity level rather than at the gene expression level and as a consequence the CCRTFs should be be identified based on their activities.

We note that the original microarray cell cycle data contains four separate experiments, corresponding to four different cell cycle synchronization method: *α*-factor arrest, temperature arrest by cdc15 mutant, temperature arrest by cdc28 mutant, and elutriation, respectively. Our analysis indicates that the *α*-factor arrest data achieves the highest quality, since the average periodogram for the inferred TF activity profiles from the other three data sets [see Additional File 1] exhibit one or multiple un-expected peaks, which may result from noise or the effect of synchronization treatment to the TF activities. Therefore, in this paper we focus our analysis on the microarray cell cycle data synchronized by *α*-factor arrest.

### Cell cycle regulated TFs

We identify 42 CCRTFs that are periodically activated in a cell cycle dependent manner at the 0.01 significance level, among which 23 have been experimentally verified according to previous studies [2,22-24]. The detail information for these CCRTFs is listed in Table 1. As shown, these CCRTFs include the well established cell cycle regulatory transcription factors: Mbp1, Swi4, Swi6, Mcm1, Fkh2 and Ndd1 [25].

**Table 1:** Cell cycle regulated transcription factors in yeast

**TF**^*a*^	**Phase**^*b*^	**p-value**	**q-value**	**Average AC score**					**k**^*c*^	**Tsai**^*d*^	**Function**
				**M/G1**	**G1**	**S**	**G2**	**M**			
ABF1		2.0E-05	5.5E-04	-3.5	**-7.0**	1.6	**9.2**	5.5	7	no	ARS-Binding Factor 1
**ASH1**		6.0E-04	0.0055	-4.0	**10.4**	5.7	6.2	**-9.1**	3	no	inhibitor of HO transcription
BAS1		0.0011	0.0074	-0.3	**-4.0**	-2.3	1.6	**5.3**	7	yes	purine and histidine biosynthesis
DAL80		1.3E-04	0.0019	**4.1**	-4.1	-4.6	**-5.4**	2.1	0	no	nitrogen degradation
**FKH2**	M	7.8E-04	0.0064	-9.1	**-11.4**	-4.6	16.4	**18.1**	7	yes	activation of M phase specific target genes
FZF1		9.9E-04	0.0072	0.0	**-7.6**	-1.9	0.0	**6.3**	7	no	sulfite metabolism
GAT3		9.9E-04	0.0072	-2.2	**14.1**	1.5	-6.9	**-7.7**	3	yes	unknown
**GCR2**		5.0E-05	9.0E-04	-2.9	**-8.1**	5.0	5.1	**6.9**	6	no	glycolysis regulatory protein
HAP2		1.6E-04	0.0020	-2.5	**-6.0**	2.8	3.4	**5.0**	6	no	global regulator of respiratory gene expression
HAP3		0.0077	0.035	2.1	**-7.6**	-2.6	-1.1	**5.7**	8	no	global regulator of respiratory gene expression
**HIR1**	S	8.0E-04	0.0064	**-10.7**	-1.2	**13.5**	7.0	4.8	5	yes	histone Regulation
**HIR2**	S	2.3E-04	0.0027	**-12.5**	-0.3	**16.5**	9.6	4.3	5	yes	histone Regulation
**HIR3**	S	5.0E-05	9.0E-04	**-9.9**	-1.4	**13.8**	7.7	2.7	5	no	histone Regulation
IFH1		0.0075	0.035	0.8	**-9.3**	-4.7	-7.4	**9.0**	8	no	regulate silencing at telomeres and HM loci
**KSS1**		8.1E-04	0.0064	0.6	-6.1	**-6.2**	-2.0	**6.2**	8	no	filamentous growth and pheromone response
**MBP1**	G1, S	0	2.0E-04	-10.6	**22.2**	11.7	-1.8	**-17.4**	3	yes	cell cycle regulation from G1 to S phase
**MCM1**	G2, M	0.0021	0.011	1.5	**-7.9**	-7.8	7.9	**15.6**	8	yes	activator of G2 and M phase-specific
MET32		3.0E-05	6.4E-04	-0.6	**-6.2**	-0.3	**5.2**	5.0	7	no	methionine biosynthetic
**MET4**		0.0019	0.011	**-8.0**	-2.3	11.4	**12.7**	-3.1	5	yes	regulator of the sulfur amino acid pathway
MSN1		6.2E-04	0.0055	**-4.4**	1.7	**5.5**	3.7	0.2	5	no	invasive growth;; hyperosmotic response
**NDD1**	M	5.3E-04	0.0052	-1.8	**-13.3**	-7.9	15.8	**23.2**	7	yes	activator of a set of late-S-phase-specific genes
NRG1		0.0035	0.018	2.4	-3.8	-3.8	**-5.5**	**4.8**	8	no	glucose repression; regulates a variety of
**OTU1**	M/G1	0.0019	0.011	0.8	**-9.7**	-4.2	-0.7	**9.8**	7	no	may contribute to regulation of protein
**PHO2**		1.1E-04	0.0019	**-3.1**	**-3.1**	2.0	3.8	**4.0**	6	no	phosphate metabolism
**REB1**		0	8.0E-05	-2.0	**-7.4**	1.5	6.5	**7.7**	7	no	RNA polymerase I enhancer binding protein
RGM1		0.0063	0.031	-0.7	**10.3**	0.0	-6.0	**-8.4**	2	no	putative transcriptional repressor
**RME1**		3.0E-05	6.4E-04	**-5.0**	-2.7	**5.9**	5.3	0.6	5	no	promotes mitosis ; sporulation
SPT2		0.0022	0.012	**-5.2**	**-5.2**	5.1	**6.7**	4.5	6	no	interact with histones and SWI-SNF components
**SRD1**	M/G1	0.0084	0.035	1.0	**-10.3**	-0.3	0.1	**7.7**	7	no	rRNA processing
**STP4**	G2/M	1.4E-04	0.0019	3.2	**6.0**	-3.7	**-5.4**	-0.6	1	no	has similarity to Stp1p, Stp2p, and Stp3p
**SWI4**	G1, S	0	5.0E-05	-16.4	**19.6**	16.2	11.0	**-17.6**	4	yes	regulate gene expression of G1 specific
**SWI5**	G1	0.0054	0.028	9.5	**13.0**	-6.7	**-11.9**	-9.3	2	yes	activates expression of early G1-specific genes
**SWI6**	G1, S	0	5.0E-05	**-16.8**	**19.8**	15.8	11.0	**-16.8**	4	yes	regulate transcription at the G1/S transition
TBS1		4.8E-04	0.0049	**-3.7**	-3.0	**6.8**	6.3	2.0	6	no	unknown
**TYE7**	G1/S	0.0098	0.039	0.1	**-7.5**	-2.3	1.0	**5.6**	7	no	putative activator in Ty1-mediated gene
**UGA3**		0.0013	0.0086	**3.1**	-1.5	-4.8	**-6.2**	2.1	0	no	GABA-dependent induction of GABA genes
UPC2		0.0015	0.0092	0.9	**-9.0**	-3.2	-0.7	**8.5**	7	no	sterol regulatory element binding protein
YAP7		0.0017	0.010	0.0	**-7.7**	-2.8	0.0	**7.4**	7	no	putative basic leucine zipper transcription
YER184C		0.0093	0.038	**4.7**	1.9	**-7.6**	-6.8	2.3	0	no	putative zinc cluster protein
YGR067C		0.0063	0.031	2.7	**-5.7**	-4.3	-4.1	**6.4**	8	no	unknown
**YOX1**	M/G1	2.5E-04	0.0027	3.5	-3.8	**-7.5**	-6.9	**7.2**	0	yes	repress ECB (early cell cycle box) activity
YPR196W		1.3E-04	0.0019	2.9	**4.0**	-4.3	**-7.7**	-0.3	1	no	putative maltose activator

a: known CCRTFs are shown in bold; b: known phases of TFs; c: inferred phases for TFs by Formula (6); d: whether identified as CCRTFs in [13]

**Figure 2:**
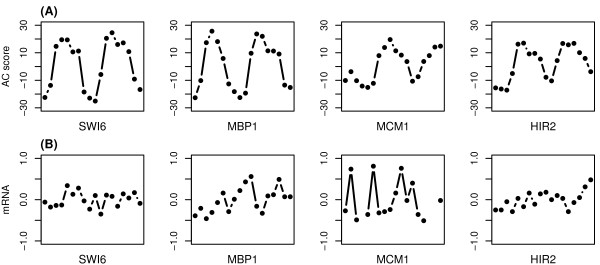
Inferred AC score profiles (A) and mRNA expression profiles (B) of 4 known CCRTFs.

Figure 2 demonstrates the inferred activity profiles and the original gene expression profiles of four CCRTFs identified by our method. In these examples, the activity profiles exhibit an apparent signature of periodicity across the cell cycle, whereas at the gene expression level the periodicity can not be detected. According to our permutation results, an AC score greater than 10 or less than -10 suggests a strong evidence of TF activation or repression. As shown, the activities of these four TFs vary dramatically during the cell cycle, indicating that they may play important regulatory roles in certain stages of the cell cycle. However, their expression changes in the original microarray data are often neglectable and do not show substantial variation across the cell cycle.

**Figure 3:**
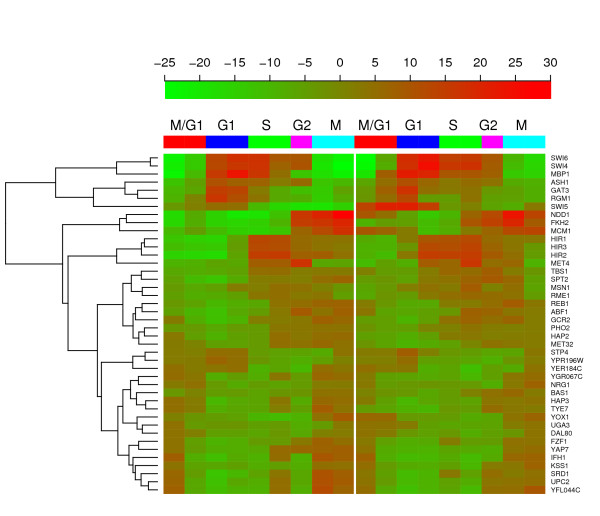
Hierarchical clustering of the 42 identified CCRTFs accoring to their AC score profiles.

In the 42 CCRTFs identified by our method, 19 have not been experimentally validated to be cell cycle regulated. But several of them have been reported as putative cell cycle TFs in several computational studies. For example, Bas1 and Gat3 have been identified as cell cycle TFs by Tsai *et al*. [13]; Abf1, Gat3, and Nrg1 have been identified by Wu *et al*. [18]; Hap2 has been identified by Yang *et al*. [14]; Bas1 and Spt2 have been identified by Cokus *et al*. [17]. Further investigation of these TFs might provide new insight into the mechanisms about how the cell cycle is regulated or how the cell cycle regulates other cellular processes. The yeast cell cycle can be divided into five different phases: M/G1, G1, S, G2 and M. In the microarray cell cycle data, each of the five stages corresponds to 4 (M/G1, G1, S, and M) or 2 (G2) time points. We calculate the average AC scores of each TF over the time points corresponding to a common phase as shown in Table 1. The phase-specific AC scores reflect the cell cycle behavior of a TF. For example, our results indicate that Fkh2 achieves the highest activity at M phase (average AC score is 18.1) and the lowest activity at G1 phase (average AC score is -11.4); Hir2 achieves the highest activity at S phase (average AC score is 16.5) and the lowest activity at M/G1 phase (average AC score is -12.5); Mbp1 achieves the highest activity at G1 phase (average AC score is 22.2) and the lowest activity at M phase (average AC score is -17.4). All these inferences are consistent with the well established knowledge that Fhk2, Hir2 and MBP1 are M phase, S phase and G1, M phase specific transcription factor, respectively. Moreover, we also estimate the active phase of each CCRTF using the statistical method described in subsection "Phase estimation" of the "Method". These 42 CCRTFs are categorized into 9 groups: *k *= 0, 1,...,8. The groups *k *= 0, 1, the groups *k *= 2, 3, the groups *k *= 4, 5, the group *k *= 6, and the groups *k *= 7, 8 achieve maximum activity at phase M/G1, G1, S, G2 and M, respectively. The statistically estimated phases for these 42 CCRTFs are also consistent with the established knowledge about these transcription factors. We apply clustering analysis to the inferred activity profiles of the 42 CCRTFs using the hierarchical clustering method [26]. As shown in Figure 3, the TFs that are activated in common phases of the cell cycle tend to be clustered together. For example, previous studies indicate that the complex SBF formed by Swi4 and Swi6 and the complex MBF formed by Swi6 and Mbp1 regulate the expression of late G1 genes [25,27,28]; Mcm1, together with Fkh1 or Fkh2, recruits the Ndd1 protein in late G2, and controls the transcription of G2/M genes [25,28,29]. Consistently, as shown in Figure 3, Swi4, Swi6 and Mbp1 follow into the same cluster that exhibits the highest activity at phase G1; Mcm1, Fhk2 and Ndd1 follows into the same cluster that exhibit the highest activity at phase M. Other than the hierarchical clustering, the above described phase estimation inherently provides a clustering method for the 42 CCRTFs, which categorize them into 9 groups: *k *= 0, 1,...,8. The image presentation of these 9 groups is shown in the supplementary documents [see Additional File 2].

Our method fails to identify several known cell cycle TFs, including Ace2 and Fkh1. It turns out that the activities of Ace2 does fluctuate periodically across the cell cycle (q-value = 0.010), but the period calculated by the periodogram is different from the genuine period of the cell cycle. For the Fkh1, although its activity profile shows an obvious fluctuation in each of the two cell cycles [See Additional File 3], the activities in the two cell cycles are not consistent with each other and thereby it fails to pass the Fisher's G-test (q-value = 0.19). This may be caused by noise or by the influence of synchronization treatment to the activity of transcription factors, which we will discuss in more detail in the next section.

### Influence of the cell cycle synchronization methods

**Figure 4:**
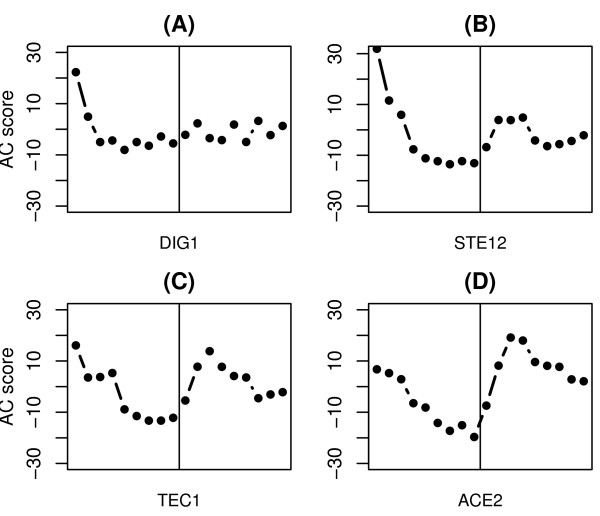
AC score profiles of four TFs which may be perturbed in activity by *α*-factor synchronizing treatment.

In order to measure gene expression during the cell cycle, the yeast cells must be synchronized using certain techniques, such as *α*-factor arrest and temperature arrest. However, these synchronization techniques may perturb the cell status and result in activity modification of TFs [30]. As a consequence, the periodic activity fluctuation of some CCRTFs may be perturbed and can not be detected. Figure 4 shows the effect of the *α*-factor to the activity of four different TFs. As shown in Figure 4A–C, Dig1, Ste12 and Tec1 exhibit extraordinary high activities at the initiation of the time series after releasing from the *α*-factor arrest (AC scores are 22.3, 31.9 and 16.1, respectively). For Ace2, although its activity is only moderately up-regulated at the initiation of the time series (AC scores is 6.8), the periodicity of its activity profiles is perturbed by the *α*-factor treatment. As shown in Figure 4D, the activity profile of Ace2 exhibits quite different patterns in the two consecutive cell cycles. In fact, Dig1, Ste12 and Tec1 are transcription factors that are activated by the MAP kinase signaling cascade and involved in the regulation of genes in mating or pseudohyphal/invasive growth pathways [31-33]. *α*-factor pheromone is the activator of the MAP kinase pathway [34], so it is not surprising to see the up-regulation of Dig1, Ste12, and Tec1 by the *α*-factor treatment.

According to previous studies, Ste12, Tec1, and Ace2 are all regulated in a cell cycle dependent manner. However, the *α*-factor treatment used to synchronize the cell cycle perturbs the periodic activity fluctuation of these TFs in the cell cycle time series, and as a consequence these CCRTFs can not be identified by our method. The statistical method proposed by Tsai *et al*. may avoid this problem, since it is not based on the test of periodicity [13]. According to their method, a TF is reported to be cell cycle regulator if the activity of the TF does not keep constant in the cell cycle and there exists at least one phase in the cell cycle where the TF is significantly up- or down-regulated. This method may result in some false positive findings. For example, the cell cycle regulator Dig1 identified by Tsai et al is obvious not a CCRTF according to its activity profile shown in Figure 4A.

### Cell cycle associated regulatory motifs

TF activities in the cell cycle can be inferred by integrating the microarray expression data with systematic ChIP-chip data. For most species, however, the large-scale ChIP-chip data is still not available. In this case, how to identify the CCRTFs? Here we provide an alternative strategy by identifying the cell cycle associated regulatory motifs. We first identify all the putative motifs that are significantly enriched in the promoter regions of all yeast genes. By integrating the systematic motif discovery data with the microarray cell cycle data, we calculate the AC score for each motif at each time point of the microarray cell cycle data. Suppose that there exists a DNA binding protein (e.g. a TF) for each motif, the AC score of a motif reflects the activity of its DNA binding protein at a time point. We define a motif as cell cycle associated regulatory motif, if its AC score profile fluctuates periodically across the cell cycle.

**Figure 5:**
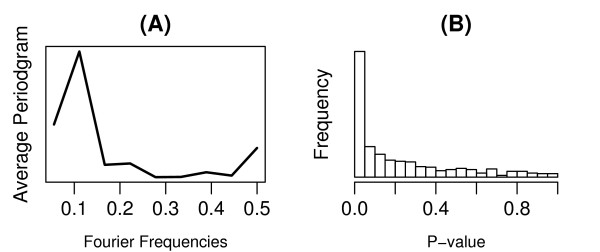
**Periodicity of inferred AC score profiles for 537 putative motifs**. (A) shows the average periodogram and (B) shows the distribution of their p-values from the Fisher's G-test.

**Figure 6:**
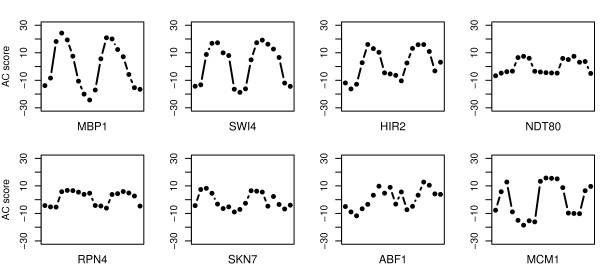
AC score profiles for 8 cell cycle associated motifs with known TFs identified by our analysis.

We identify 537 putative motifs and calculate their AC score profiles in the cell cycle. Figure 5A shows the average periodogram for the AC score profiles of these 537 motifs. As shown, it exhibits a dominate peak at the Fourier frequency 0.11, the genuine cell cycle frequency. We test the significance of periodicity for these profiles and the distribution of their p-values is shown in Figure 5B. Among these 537 putative motifs, 97 are found to be cell cycle associated at the 0.01 significance level according to our analysis (FDR = 0.04). In these 537 putative motifs, 46 can be associated with known TFs according to previous literatures, including 12 cell cycle TFs: Ace2, Fkh1, Hir2, Mbp1, Mcm1, Ndt80, Rpn4, Skn7, Ste12, Swi4, Ume6, and Xbp1. In the 46 motifs with known TFs, 8 are found to be cell cycle associated motifs according to our results. These 8 motifs are known to be regulated by Mbp1, Swi4, Hir2, Ndt80, Rpn4, Skn7, Abf1, and Mcm1, respectively. Among them, 7 are experimental verified cell cycle TFs and the other one, Abf1, is reported to be cell cycle TF in the computational analysis performed by Wu *et al*. [18]. Detail information for these motifs is listed in the supplementary documents [see Additional File 4] and the inferred activity profiles of them are shown in Figure 6. Note that we miss 5 cell cycle TFs: Ace2, Fkh1, Ste12, Ume5, and Xbp1. Our analysis indicates that the activity of Ace2 fluctuates periodically across the cell cycle (p-value = 0.0011), but the estimated Fourier frequency is not equal to the cell cycle frequency. This is consistent with the result obtained using ChIP-chip data. For the other four missed cell cycle TFs: Fkh2 (p-value = 0.025), Xbp1 (p-value = 0.015), Ste12 (p-value = 0.076), and Ume6 (p-value = 0.067), the p-values from periodicity test are relatively small, although they do not pass our criteria. Overall, these results suggest that our method is able to identify CCRTFs with high accuracy by integrating microarray expression data with the motif discovery data from pure in-silico sequence analysis.

## Discussion

To test whether a TF is cell cycle regulated, Tsai *et al*. examine if there exists at least one phase in the cell cycle where the TF is significantly activated by comparing the expression levels of its target genes with those of non-target genes [13]. In this paper, we propose a two-step method: infer the activity profiles of a TF in the cell cycle and then test the significance of its periodicity. In comparison with the method suggested by Tsai *et al*., our method has the following advantages: (1) it need not to pre-define the target and non-target gene sets for each TF based on more or less arbitrarily selected threshold setting and is thereby easier to be implemented; (2) it deals with TF activity perturbation caused by synchronization treatment in a more conservative manner, and thereby avoid some false positive findings; (3) it can detect the CCRTFs that are periodically but moderately activated during the cell cycle. On the other hand, there is also a limitation for this method: synchronization treatments may perturb the periodic activity fluctuation of some TFs and result in false negatives. In order to achieve more accurate results, it may be useful to combine these two methods. For example, after the implementation of our method, we can further examine the AC scores of those non-CCRTFs at the initial time point. If the initial AC score of a TF is significantly high or low, its activity may be perturbed by the synchronization treatment. We therefore exclude one or more initial time points and apply the Tsai *et al*.'s method to the remaining time points to examine again whether it is cell cycle regulated.

Several methods have proposed to infer the TF activities from microarray expression data by integrating it with ChIP-chip data or motif information [35-40]. All these methods assume a linear relationship between gene expressions and TF-gene binding affinities (ChIP-chip data), motif occurrences, or motif matching-scores, which may not be valid considering the high complexity of gene transcriptional regulation. Moreover, some of these methods, such as gNCA, require a complicated pre-processing step and some constraints. In contrast, our method does not require the linear assumption and easy to be implemented. It is exciting to see that the CCRTFs can be identified with high accuracy by integrating microarray cell cycle data with the in-silico motif discovery data. Microarray cell cycle data sets are available for several species, including fission yeast, mouse and human. On the other hand, systematic ChIP-chip data are still not available for most species other than the budding yeast. Our method may be applied to the microarray cell cycle data in these species to identify the all the cell cycle associated regulatory motifs. Then experimental techniques or computational methods can be used to associate these motifs with TFs. This may lead to systematic identification of the CCRTFs in these species.

## Conclusion

In conclusion, we present a method to identify CCRTFs by integrating microarray cell cycle data with systematic ChIP-chip data or motif discovery data. For each TF, the method infers its activity profile across the cell cycle and then tests the significance of periodicity of the profile. Application of this method to the yeast microarray cell cycle data and the ChIP-chip data results in identification of 42 CCRTFs, among which 23 have been experimentally verified. Importantly, reliable results are also achieved by integrating the microarray expression data with the in-silico motif discovery data. The method provides a useful tool to investigate the cell cycle transcriptional regulation, especially in those species where large-scale ChIP-chip data are not available.

## Methods

### Microarray cell cycle data and ChIP-chip data

In this study, we utilize the microarray cell cycle data published by Spellman *et al*., which contains expression profiles of 6178 yeast genes during the cell cycle [2]. RNA samples were collected every 7 minutes after *α*-factor synchronization and hybridized with cDNA microarray, resulting in the microarray time series data with 18 time points covering two cell cycles. During hybridization, RNA from asynchronous cultures was used as control. We represent the microarray cell cycle data by a matrix *E *(referred to as expression matrix) with 6178 rows and 18 columns, each row corresponding to a gene and each column corresponding to a time point. The values in the expression matrix *E *are log ratios. In the systematic ChIP-chip experiment performed by Harbison *et al*., in vivo binding sites across the whole genome for 203 yeast TFs were identified under YPD condition [19]. Each TF-gene pair was assigned an occupancy ratio which reflects binding affinity of the TF to the promoter region of the gene. Roughly, larger occupancy ratios suggest strong binding affinities. We also represent the ChIP-chip occupancy ratio data by a matrix *B *(referred to as binding score matrix) with 6229 rows and 203 columns, each row corresponding to a gene and each column corresponding to a TF. Note that the occupancy ratios in the binding score matrix *B *are in their original scale without log transformation.

### Inferring TF activities based on the ChIP-chip data

We use the BASE method proposed by Cheng *et al*. to infer the activities of transcription factors at each time point of the cell cycle data [21]. Given an expression profile e = (e_1_, e_2_,...,e_*N*_) (e.g. a column corresponding to a specific time point in the expression matrix *E*) and a binding profile *b *= (*b*_1_, *b*_2_,...,*b*_*N*_) (e.g. a column corresponding to a specfic TF in the binding score matrix *B*), where N is the number of common genes in *E *and *B*, we infer the activity of this TF in this time point using the following method. First, we sort the expression vector *e *in the decreasing order to obtain a sorted vector *e*' = (*e*_(1)_, *e*_(2)_,...,*e*_(*N*)_). We also rearrange the binding vector *b *into *b*' = (*b*_(1)_, *b*_(2)_,...,*b*_(*N*)_) according to the gene orders in the sorted expression vector *e*'. Second, we combine the two vectors *e*' and *b*' into an increasing function *f*(*i*) defined as following:

$$f(i)=\frac{\sum_{j=1}^{i}\left|e_{\left(j\right)}b_{\left(j\right)}\right|}{\sum_{j=1}^{N}\left|e_{\left(j\right)}b_{\left(j\right)}\right|},$$

where 1 ≤ *i *≤ *N*. Meanwhile we calculate another increasing function *g*(*i*) for *e*' itself as

$$g(i)=\frac{\sum_{j=1}^{i}\left|e_{\left(j\right)}\right|}{\sum_{j=1}^{N}\left|e_{\left(j\right)}\right|}.$$

Third, we find out the *i*_*max *_that achieves the maximum deviation between *f *(*i*) and *g*(*i*), that is, $i_{max}=\underset{i=1,2,\cdots ,N}{\arg\max}\left|f(i)-g(i)\right|$. Then a pre-score is defined as

ps* = *f*(*i*_*max*_) - *g*(*i*_*max*_).

Fourth, we permute the reordered binding vector *b' K *times (*K *= 10,000), and recalculate the pre-scores by replacing *b*' in Formula (1) with each of the permutated binding vectors, resulting in a permutated pre-score vector *ps*^*perm *^= (*ps*^1^, *ps*^2^,...,*ps*^*K*^). Finally, we define an activity change score (AC score) as

$$AC=\frac{ps^{*}-MEAN(ps^{perm})}{SD(|ps^{perm}|)},$$

where *MEAN*(*ps*^*perm*^) is the mean of *ps*^*perm *^and *SD*(|*ps*^*perm*^|) is the standard deviation of the absolute values of *ps*^*perm*^. The AC score reflects the relative activity of the considered TF in the synchronized sample with respect to the non-synchronized control at this time point.

We calculate the AC scores for all the 203 TFs available from the ChIP-chip data in each of the 18 time points of the cell cycle data. As a result, for each TF we obtain an activity profile across the cell cycle, and we then perform periodicity analysis for these activity profiles. The C++ program for AC score calculation is available for download at [41].

### Periodicity analysis

We use two criteria to determine whether a TF is cell cycle regulated: (1) whether the TF activities fluctuate periodically across the cell cycle; (2) whether the period of its activity fluctuation matches the cell cycle period. To implement these two criteria, we apply the periodicity analysis techniques introduced by Wichert *et al*. to the inferred AC score data containing activity profiles for 203 yeast TFs. [8].

First, we use the average periodogram to assess the presence of TFs that are periodically expressed in the original microarray expression data or periodically fluctuate in activities in the inferred TF AC score data. The average periodogram is a simple extension of the standard periodogram. The standard periodogram is a tool to analyze a single time series: if a time series (e.g. a TF expression profile or an activity profile across the cell cycle) contains significant sinusoidal component, then the periodogram would exhibit a peak at a specific frequency. Average periodogram is the average of multiple time series with a common block size, e.g. the average of periodograms for all genes in a microarray time series data (each gene corresponds to a time series). We use average periodogram to assess the existence of periodically expressed genes in the microarray cell cycle data. If there are a few genes exhibiting strong periodicity, the periodograms of them would dominate the average periodogram and exhibit a visible peak. Since all the periodically expressed genes should have the same frequency, i.e. the cell cycle frequency, we would expect to see a single peak in the average periodogram for microarray cell cycle data. In practice, however, we may observe one or more small peaks other than the main peak, which are caused by the noise in the data. Therefore, the pattern of peaks in average periodogram can reflects the quality of microarray cell cycle data.

Second, we use the Fisher's G-test to examine whether the expression or activity of a TF behaves like a pure random process or whether it shows a periodical pattern. The significance of periodicity was tested for all the 203 inferred TF activity profiles and a p-value is assigned to each TF. Third, we calculate the q-values for those p-values using a method of FDR to correct for multiple testings. Finally, for each significant TF, we use the standard periodogram to estimate its period and compare the estimated period with the known cell cycle period. With the above described procedure, we identify 42 CCRTFs at the 0.01 significance level (FDR < 0.04) based on the above inferred AC score data. To implement these techniques, the R package GeneTS is used.

### Phase estimation

Let us model the periodic activity profile of a TF as following:

$$Y_{t}=\beta \cos(2\pi \frac{t-k}{T})+\varepsilon_{t},$$

where *β *is a positive constant, *T *represents the period, and *k *represents the phase. Since the microarray data covers exactly two cell cycles by 18 time points, *T *= 9 and the phase *k *takes an integral value from 0 to 8. The phase for a TF can be estimated using the following formula:

$$\hat{k}=\arg\underset{k}{\min}\sum_{t=0}^{17}\left(Y_{t}\cos(2\pi \frac{t-k}{T})\right).$$

We estimate the phase for each of the 42 identified CCRTFs, which basically categorized them into 9 groups. For example, according to our results, the cell cycle TF, Fkh2, belongs to the group with phase *k *= 7, which achieves the maximum activity at M phase in the cell cycle.

### Motif discovery data

In a systematic analysis performed by Beer *et al*. [20], 666 potential regulatory motifs that are significantly enriched in the promoter regions (the DNA sequences from translation initiation site up to 800 bp upstream) of all yeast genes using the AlignACE software [42,43]. The occurrences of each motif in the promoter region of each gene are then determined as those with matching-scores larger than 0.5. We download this motif discovery data from [44]. After removing redundancy, we select 539 motifs from these 666 putative motifs, in which 46 are associated with known transcriptional factors according to literatures. Based on this data, we define a matching-score matrix *M*, which contains 6328 rows each corresponding to a yeast gene, and 539 columns each corresponding to a putative motif. The element of M is the aggregated matching-score of a motif in the up-stream region of a gene, that is, the matching-scores of all the occurrences for the same motif are aggregated in case of multiple occurrences. When no occurrence is found in the upstream region of a gene, the score is set to 0. The aggregated matching-score, to some extent, reflects the binding affinity of a motif to the promoter of a gene.

### Identification of cell cycle associated motifs

For each putative motif we assume there is a TF that binds it to regulate gene expression. By integrating the microarray gene expression matrix *E *with the matching-score matrix *M*, we can infer the activity profile of the TF corresponding to a putative motif in the cell cycle. The method is similar to the one used to infer TF activity profile by integrating microarray cell cycle data with ChIP-chip data, except that the binding score matrix *B *obtained from ChIP-chip experiment is replaced by the matching-score matrix *M *obtained from in-silico sequence analysis.

We then perform the above described periodicity analysis and estimate the active phase for each imaginary TF of the putative motifs. According to the results from this analysis, we identify the cell cycle associated motifs which are potential regulatory sites for some CCRTFs.

## Availability and requirements

Project name: BASE

Project homepage: 

Operating system: Microsoft Windows XP system

Programming language: Visual C++ 6.0

Other requirements: None

License: The tool is available free of charge.

Any restrictions to use by non-academics: None

## Authors' contributions

CC designed the method, wrote the code, carried out the analysis, and drafted the manuscript. LL participated in design and coordination of the study. Both authors read and approved the final manuscript.

## Supplementary Material

Additional file 1 Average periodograms for 203 inferred TF AC score profiles in four yeast microarray cell cycle data sets. In these data sets, yeast cells are synchronized by *α*-factor arrest, temperature arrest by cdc15 mutant, temperature arrest by cdc28 mutant, and elutriation, respectively. Click here for file

Additional file 2 Visualization of inferred AC score profiles for 42 CCRTFs according to their statistical phase *k*. Click here for file

Additional file 3 Inferred AC score profiles for FKH1 across two cell cycles. Click here for file

Additional file 4 Complete information for 8 cell cycle associated yeast motifs. Click here for file
